# eCONUT-UC for nutritional screening and malnutrition identification in ulcerative colitis

**DOI:** 10.3389/fnut.2026.1907895

**Published:** 2026-09-24

**Authors:** Viridiana Montsserrat Mendoza-Martínez, Desiree López-González, Patricia Clark, Jorge Luis De-León-Rendón, Ernesto Roldan-Valadez, Guillermo Meléndez-Mier, Alma L. Almiray-Soto, Roberto Baños-Vázquez, Nallely Bueno-Hernández

**Affiliations:** 1 Proteomics and Metabolomics Laboratory, Research Division, Hospital General de México “Dr. Eduardo Liceaga” Mexico City, Mexico; 2 Faculty of Medicine, Universidad Nacional Autónoma de México (UNAM), Mexico City, Mexico; 3 Clinical Epidemiology Research Unit, Hospital Infantil de México Federico Gómez, Mexico City, Mexico; 4 Secretary of the General Health Council, Mexico City, Mexico; 5 Inflammatory Bowel Disease Clinic, Coloproctology Service of Hospital General de México “Dr. Eduardo Liceaga”, Mexico City, Mexico; 6 Division of Research, Instituto Nacional de Rehabilitación Luis Guillermo Ibarra Ibarra, Mexico City, Mexico; 7 Department of Radiology, I.M. Sechenov First Moscow State Medical University (Sechenov University), Moscow, Russia; 8 Faculty of Public Health and Nutrition (FASPyN), Universidad Autónoma de Nuevo León, Monterrey, Mexico

**Keywords:** CONUT score, diagnostic accuracy, epidemiology, GLIM criteria, inflammatory bowel disease, malnutrition, nutritional screening, phase angle

## Abstract

**Background:**

Detecting malnutrition in patients with ulcerative colitis is considered a clinical challenge. The Global Leadership Initiative on Malnutrition (GLIM) criteria constitute a standardized approach for diagnosis. However, the Controlling Nutritional Status (CONUT) score is a simple tool based on laboratory analysis; its performance in early malnutrition detection could be improved. Our aim was to exploratorily derive and assess the apparent diagnostic performance of enhanced CONUT-based scores (eCONUT-UC) for identifying GLIM-defined malnutrition in patients with ulcerative colitis.

**Methods:**

We conducted a cross-sectional study in patients with ulcerative colitis. We measured weight, height, body mass index, and body composition using DXA and BIA. We extracted recent laboratory results and clinical data from medical records. We used the GLIM criteria as the reference standard for detecting malnutrition in ulcerative colitis. We evaluated the diagnostic performance of the eCONUT-UC by incorporating phase angle (PhA), hemoglobin (Hgb), and triglycerides (TG). We calculated sensitivity, specificity, and area under the curve (AUC).

**Results:**

We included 51 patients, of whom 15 (29%) met GLIM criteria for malnutrition. The original CONUT score had a sensitivity of 73% and a specificity of 67%. The eCONUT-UC-PhA and eCONUT-UC-Hgb showed a sensitivity of 80%, with an AUC of 0.741 [95% CI: 0.587–0.895] and 0.738 [95% CI: 0.586–0.890]. The eCONUT-UC-TG showed lower diagnostic performance.

**Conclusion:**

The eCONUT-UC showed higher sensitivity in detecting malnutrition than the original CONUT. Its use as a rapid and accessible screening tool supports its potential implementation in clinical practice, particularly in resource-limited settings, to facilitate timely nutritional intervention for patients with ulcerative colitis.

## 1 Introduction

Ulcerative colitis (UC) is a chronic inflammatory bowel disease (IBD) characterized by persistent inflammation of the colon’s lining. The most common gastrointestinal symptoms include diarrhea, rectal bleeding, abdominal pain, and tenesmus, which often make food intake and dietary tolerance difficult (1). As a result, many UC patients modify their diets to manage symptoms, often without professional nutritional guidance. These unsupervised dietary changes often lead to nutritional imbalances and increase the risk of malnutrition (MN). MN is a common complication in patients with UC and has been associated with skeletal muscle mass (SMM) wasting, sarcopenia, higher hospitalization rates, poorer treatment response, a worse disease prognosis, and increased mortality. While diagnosing MN remains a challenge in clinical practice, it is often limited to settings with trained nutrition specialists. Over the past decade, several definitions and tools for identifying MN have been proposed; most are nutritional risk screening tools rather than diagnostic tools. The latter combines phenotypic criteria, such as weight loss, a low body mass index (BMI), and a reduced muscle mass index (MMI), with etiological factors such as decreased dietary intake or inflammation (2, 3).

There has been significant progress in standardizing MN diagnosis. The GLIM criteria require clinical assessments of body composition, diet, and inflammation, which may limit their routine use in outpatient settings.

Malnutrition in the UC is also commonly characterized by alterations in body composition, including decreased phase angle (PhA), weight loss, and a normal or elevated body mass index (BMI). However, MN is not only reflected in anthropometric or body composition parameters; biochemical alterations can also occur, such as changes in serum albumin, hemoglobin (Hgb), total cholesterol, triglycerides (TG), and lymphocyte count, especially in the early stages of nutritional deterioration and preceding evident changes in body composition. These changes in biochemical parameters may reflect a combination of factors, including inflammation, reduced food intake, altered metabolism, and immune dysfunction, highlighting the importance of tools that can identify MN early (4, 5).

The CONUT score integrates serum albumin, total cholesterol, and total lymphocyte count to provide an objective assessment of nutritional and immunological status. In several studies, CONUT demonstrated good agreement with comprehensive nutritional assessment methods that include anthropometric, biochemical, clinical, and dietary components (6). Because the CONUT score relies exclusively on routinely available laboratory parameters, it is a potentially attractive and practical tool for detecting MN in clinical settings where comprehensive dietary assessment is not readily available.

In this context, this study aimed to exploratorily derive and assess the apparent diagnostic performance of enhanced CONUT-based scores (eCONUT-UC) for identifying GLIM-defined malnutrition in patients with ulcerative colitis. The proposed scores should be considered candidate screening tools requiring subsequent internal and external validation.

## 2 Materials and methods

### 2.1 Study design

This was a cross-sectional diagnostic study conducted at the Hospital General de México “Dr. Eduardo Liceaga” (HGMEL), between 2023 and 2026. The study’s participants flowchart (Figure 1) follows the Standards for Reporting Diagnostic Accuracy Studies (STARD) guidelines, and the corresponding checklist is provided as supplementary material (Supplementary Figure 1) (7).

**FIGURE 1:**
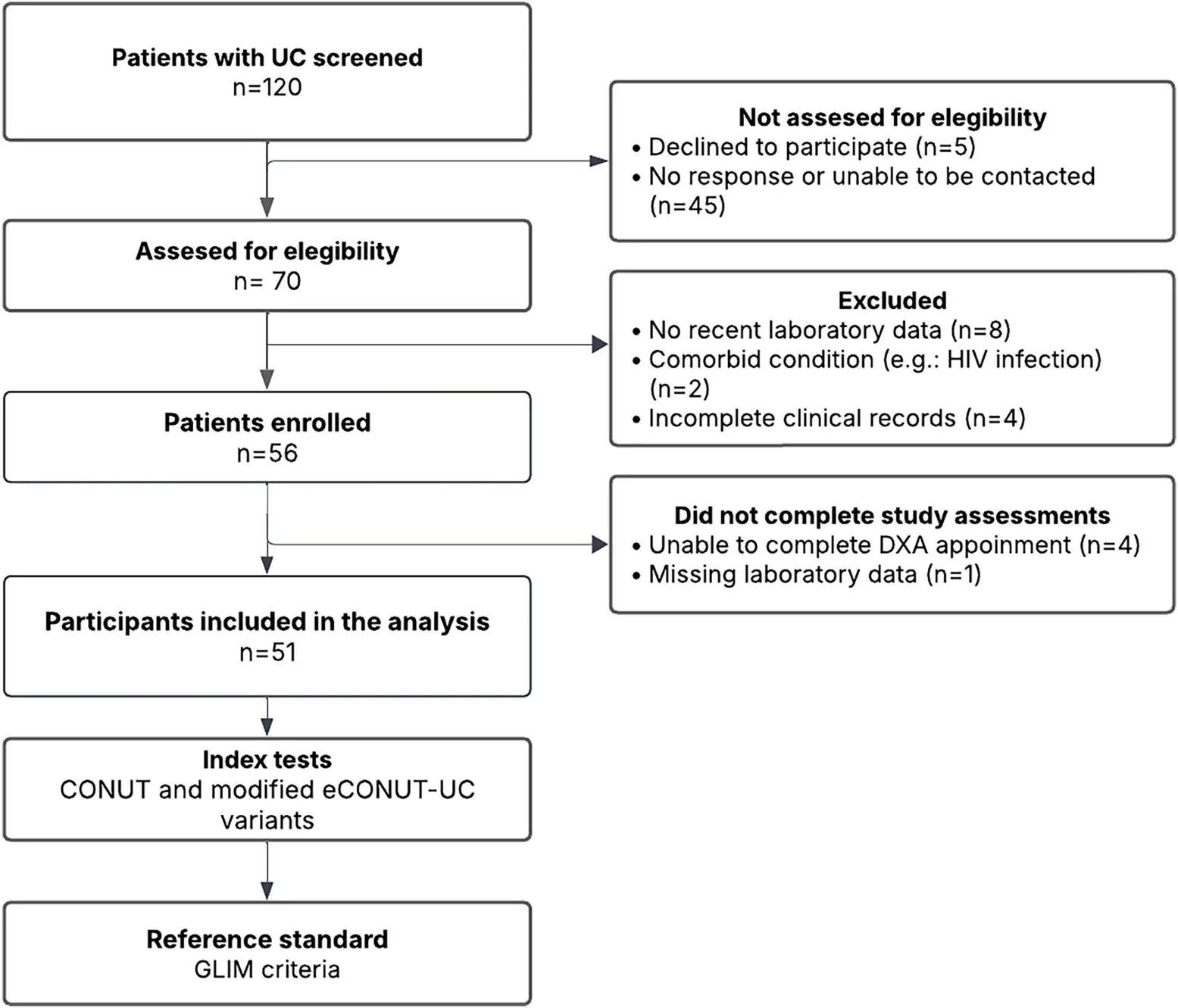
STARD flowchart. UC, ulcerative colitis; DXA, dual-energy X-ray absorptiometry; HIV, human immunodeficiency virus; n, number of participants.

### 2.2 Study population

We recruited ambulatory UC patients with a confirmed diagnosis of UC in a consecutive series, as established by a gastroenterologist. Both genders, aged over 18 years. Comorbidities such as type 2 diabetes mellitus, hypertension, and stable thyroid disease were not considered exclusion criteria and were permitted in the study population. Exclusion criteria were the presence of pacemakers or limb amputations; patients who did not have the required laboratory tests performed at the study hospital within 2 weeks of the clinical and nutritional assessment, or had incomplete clinical records that precluded application of the GLIM criteria or calculation of the CONUT score; patients with cancer who required other kinds of treatment; pregnancy; or lactation.

### 2.3 Ethical considerations

This study was conducted in accordance with the ethical principles established in the Declaration of Helsinki and the ethical and research guidelines of the HGMEL. This project has an approval number of DI/23/501/03/31 and is registered with ClinicalTrials.gov under the identifier NCT06648551 after participant recruitment had commenced and was therefore retrospectively registered. All participants provided written informed consent before enrollment.

### 2.4 Clinical and disease-related variables

Patients underwent a gastrointestinal symptom assessment, and stool frequency and consistency were recorded according to the Bristol Stool Scale (8). The presence of rectal bleeding or mucus was also noted. Vital signs, including blood pressure, body temperature, and oxygen saturation, were also recorded, along with comorbidities and current medications, including corticosteroids and biologic therapy.

Disease activity was assessed using the modified Truelove and Witts (TLW) criteria to classify patients as active or in remission (9). Disease extent was obtained from the medical history and categorized as E1 (proctitis), E2 (left-sided colitis), or E3 (pancolitis) (10).

### 2.5 Nutritional assessment

#### 2.5.1 Anthropometric assessment

Body weight (kg) and height (m) were measured using a calibrated SECA^®^ 284 scale and stadimeter (Seca GmbH & Co., Hamburg, Germany), and a stadiometer of the same (SECA direct print 284 scale stadiometer), and BMI was calculated as kg/m^2^. Measurements were obtained with participants wearing light clothing and no shoes.

#### 2.5.2 Body composition assessment

##### 2.5.2.1 DXA

The body composition assessment was performed using a Lunar-iDXA densitometer (GE Healthcare^®^, Madison, WI, USA), according to the manufacturer’s instructions (11). Scans were analyzed using ENCORE^®^ software (version 15). We performed daily phantom calibration.

The 3-component body composition was reported for total body and by regions (arms, trunk, and legs): Fat mass (FM), Lean Mass (LM), and Bone Mineral Content (BMC); however, only FM and LM were included in the present analysis. Appendicular lean mass (ALM) was calculated as the sum of lean soft tissue mass from both arms and legs. ALM was adjusted for height squared to obtain the appendicular lean mass index (ALMI, kg/m^2^). Reduced muscle mass was defined according to the GLIM cut-off points, using an ALMI < 7.0 kg/m^2^ for men and <5.5 kg/m^2^ for women (12).

All DXA scans were performed by personnel certified by the International Society for Clinical Densitometry (ISCD) (13).

##### 2.5.2.2 Bioimpedance

We performed bioelectrical impedance analysis (BIA) using the RJL Quantum IV system (RJL Systems Inc., Clinton Township, MI, USA). Measurements were obtained under standardized conditions, with the participant supine, after fasting for at least 8 h, and after avoiding intense physical activity for 24 h before assessment.

Bioelectrical impedance analysis estimates PhA, fat mass percent (FM%), and SMM, using the formula for Mexican adults (14).

The patient was instructed to remove any metal objects and to lie supine with the limbs separated at 45°. The areas where the 4 electrodes were placed were previously cleaned with alcohol (under the metacarpal phalanx in the middle of the dorsum of the hand and just below the transverse arch, on the dorsal side of the foot, immediately behind the second metatarsal phalanx). The cables were placed, and the participant’s reading was taken. At the end of the measurement, the data were entered into the online analyzer: https://apps.rjlsystems.com/app/interactive/.

This procedure generated a PDF format that was used to extract the data to the database.

#### 2.5.3 Dietary assessment

Dietary intake was assessed using 24-h dietary recalls (24R). Trained personnel conducted face-to-face interviews. Participants were questioned about all foods and beverages consumed in the previous 24 h, including ingredients and preparation methods. The 24R were processed using EvalFINUT^®^ software version 2.0 (FINUT, Spain) to determine total energy and macronutrient intake. Energy requirements (ER) were calculated based on current weight, following clinical nutrition guidelines (25–30 kcal/kg/day) (15).

Using dietary intake data from EvalFINUT^®^ and the estimated ER, reduced intake was defined as less than 50% of the ER. This criterion was used to satisfy the etiologic requirement of the GLIM criteria (16).

#### 2.5.4 Biochemical assessment

Laboratory data were obtained from electronic medical records; only laboratory tests performed at the reference hospital within 2 weeks of the clinical and nutritional assessment were included. Mandatory parameters included complete blood count, serum albumin g/dl, total cholesterol, potassium, and C-reactive protein (CRP, mg/L), Erythrocyte Sedimentation Rate (ESR, mm/h). Additional biochemical variables, such as glucose (mg/dl), triglycerides (TG, mg/dl), creatinine (mg/dl), uric acid (mg/dl), urea (mg/dl), high-density lipoprotein cholesterol (HDL-c, mg/dl), low-density lipoprotein cholesterol (LDL-c, mg/dl), alanine aminotransferase (ALT, U/L), aspartate aminotransferase (AST, U/L), gamma-glutamyl transferase (GGT, U/L).

### 2.6 Definition of malnutrition

To detect MN using the CONUT score and GLIM criteria, available clinical and laboratory data were used; however, no formal blinding was performed between assessments.

#### 2.6.1 GLIM criteria

The GLIM criteria were prespecified as the reference standard for detecting MN in patients with UC. Phenotypic criteria included: BMI < 20 kg/m^2^, self-reported unintentional weight loss in the last 6 months, and reduced muscle mass assessed as ALM by DXA. Low muscle mass was defined as ALMI < 5.5 kg/m^2^ for women and <7 kg/m^2^ for men (2).

Etiological criteria included reduced dietary intake to <50% of estimated ER, based on 24R; presence of active disease according to TLW criteria; or evidence of inflammation, defined as CRP > 5mg/L.

Patients meeting at least one phenotypic and one etiological criterion were classified as MN (see Supplementary Table 1) (2).

#### 2.6.2 CONUT score

After obtaining laboratory results from the electronic medical record, we calculated the CONUT. We classified patients by score into normal nutritional status (0–1 points), mild MN (2–4 points), moderate MN (5–8 points), and severe MN (9–12 points). We considered MN positive when the total score was ≥2 points (6) (see Supplementary Table 2).

#### 2.6.3 Determination of cut-off points for the eCONUT-UC and construction of ordinal variables

To identify candidate variables for exploratory modification of the CONUT score, we examined correlations between the original CONUT score and anthropometric, biochemical, clinical, and dietary variables. Phase angle (PhA), hemoglobin (Hgb), and triglycerides (TG) showed the strongest statistically significant correlations with CONUT and were therefore selected as candidate variables. Importantly, we did not use GLIM-defined malnutrition during this initial candidate-variable identification step.

Subsequently, GLIM-defined malnutrition was used as the reference standard in ROC analyses to derive exploratory cut-off points for PhA, Hgb, and TG. We selected cut-offs based on sensitivity and specificity along the ROC curve, with greater emphasis on sensitivity because the intended purpose of eCONUT-UC is nutritional screening. Because these thresholds and their subsequent diagnostic performance were evaluated in the same cohort, they should be regarded as derivation-sample thresholds and the resulting performance measures as apparent estimates requiring validation in independent data. The modified scores are as follows: normal (0–1 point), mild MN (2–6 points), moderate MD (7–10 points), and severe MN (11–15 points), as shown in Supplementary Table 3. We considered it positive for MN when the total score was ≥2 points.

### 2.7 Statistical analysis

The sample size was calculated for the primary purpose of assessing the diagnostic performance of the CONUT score and not specifically for the exploratory development of the eCONUT-UC variants. A ratio of 94% was used for Ps and 62% (2), for P, with a confidence level of 95%, a margin of error of 5%, and a statistical power of 80%, resulting in a total of 56 patients. The development and evaluation of the eCONUT-UC variants were not pre-established in the original ClinicalTrials.gov record and were therefore carried out as exploratory post-hoc, hypothesis-generating analyses.

We performed the Shapiro-Wilk test to assess variable normality. Descriptive statistics were used to report baseline data using measures of central tendency and dispersion. An independent-samples *t*-test was performed to assess differences in patients with MN between CONUT and GLIM. We excluded participants without laboratory data or DXA-derived body composition measurements from the analysis.

Because MN, as measured by GLIM criteria, is a categorical outcome, GLIM was not used to initially identify candidate variables; however, GLIM-defined malnutrition was subsequently used as the reference standard for ROC-based derivation of candidate cut-off points and for estimating their apparent diagnostic performance. To identify candidate variables for the eCONUT-UC versions, we performed Pearson correlation analyses between quantitative variables from anthropometric, biochemical, clinical, and dietary measurements and the CONUT score. PhA, Hgb, and TG were highlighted. Significant inverse correlations were observed for PhA (*r* = −0.419, *p* = 0.002; *n* = 50), Hgb (*r* = −0.417, *p* = 0.002; *n* = 51), and TG (*r* = −0.383, *p* = 0.006; *n* = 50).

To evaluate the diagnostic performance of the CONUT score and the enhanced CONUT-derived scores (eCONUT-UC-PhA, eCONUT-UC-Hgb, and eCONUT-UC-TG), sensitivity, specificity, positive predictive value (PPV), negative predictive value (NPV), and Youden Index were calculated using GLIM-defined MN as the reference standard. Agreement between CONUT-based classifications and GLIM diagnosis was assessed using Cohen’s kappa coefficient. AUCs were compared pairwise between the original CONUT and each eCONUT-UC variant using DeLong’s test for correlated ROC curves. Analyses used the available cases for each pairwise comparison.

Analyses of diagnostic performance were conducted using the available cases for each eCONUT-UC variant. One PhA value and one TG value were missing; therefore, the analyses for eCONUT-UC-PhA and eCONUT-UC-TG included 50 participants. The 2 × 2 table is available in the supplementary tables (see Supplementary Table 4).

We used IBM SPSS Statistics version 25 (IBM, Armonk, NY, USA) for the analysis, setting *p* < 0.05 as the criterion for significance.

## 3 Results

A total of 120 patients were invited to participate; of these, 56 met the inclusion criteria. These patients were scheduled for body composition assessment using DXA and BIA, as well as 24R evaluation or application. For the final analysis, 5 patients were excluded: 4 because the DXA appointment could not be scheduled, and 1 because their medical records did not contain recent laboratory results (Figure 1).

### 3.1 Demographic results

After database curation, we analyzed 51 patients. 59% were women and 41% were men, with an average age of 42 ± 14 years. Regarding disease extent, 16% had proctitis, 24% had left colitis, 55% had pancolitis, and 5% had an uncertain extent. Their average BMI was 25 ± 4.4 kg/m^2^.

Regarding current symptoms, 20% reported blood in their stool, 31% reported mucus in their stool, and 41% reported joint pain. Regarding medication use, 92% of patients were taking 5-aminosalicylic acid (5-ASA), 20% had used steroids in the last 3 months, and 29% were receiving biologic therapies. As for disease activity, 55% were in remission, while 45% had mild activity, according to TLW criteria as detailed in Table 1.

**TABLE 1:** Demographic and clinical characteristics of patients with UC.

Characteristic	UC (*n* = 51)
Age at diagnosis, years (mean ± SD)	35 ± 15
BMI, kg/m^2^ (mean ± SD)	25 ± 4.4
Age, years (mean ± SD)	42 ± 14
Sex, n (%)	
Women	30 (59)
Men	21 (41)
Extent of the UC, n (%)	
Proctitis	8 (16)
Left colitis	12 (24)
Pancolitis	28 (55)
Uncertain	3 (5)
Current symptoms, n (%)	
Blood in bowel movements	10 (20)
Mucus in bowel movements	16 (31)
Joint pain	21 (41)
Medicines, n (%)	
5-ASA	47 (92)
Steroids	10 (20)
Biologicals	15 (29)
Disease activity, n (%)	
Active	23 (45)
Remission	28 (55)
Laboratory (mean ± SD)	
Glucose, mg/dl	91.6 ± 11.6
Urea, mg/dL	28.5 ± 7.2
Creatinine, mg/dL	0.7 ± 0.1
Uric acid, mg/dL	4.7 ± 1.1
Cholesterol total, mg/dl	172.1 ± 41.8
Triglycerides, mg/dl	121.2 ± 63.5
HDL-c, mg/dl	51.6 ± 9.7
LDL -c, mg/dl	106.6 ± 40.8
Albumin, g/dl	4.3 ± 0.3
ALT, U/L	24.2 ± 17.5
AST, U/L	24.3 ± 15.1
GGT, U/L	48.4 ± 86.3
CRP, mg/L	9.9 ± 10.5
ESR, mm/h	17.2 ± 19.3
Leukocytes, ×10^3^/μL	6.2 ± 1.9
Erythrocytes, ×106/μL	4.6 ± 0.5
MCV, fL	88.9 ± 7.1
MCH, pg	30.0 ± 3.1
Platelets, ×10^3^/μL	287 ± 98
Hemoglobin, g/dl	13.8 ± 2
Hematocrit, %	41.2 ± 5.1

UC, ulcerative colitis, SD, standard deviation; BMI, body mass index; 5-ASA, 5-aminosalicylic acid; n, number of patients; HDL-c, high-density lipoprotein cholesterol; LDL-c, low-density lipoprotein cholesterol; ALT, alanine aminotransferase; AST, aspartate aminotransferase; GGT, gamma-glutamyl transferase, CRP, C-reactive protein; ESR, Erythrocyte Sedimentation Rate; g/dl, gram per deciliter.

### 3.2 Baseline biochemical characteristics

The biochemical values were glucose 91.6 ± 11.6 mg/dl, urea 28.5 ± 7.2 mg/dl, creatinine 0.7 ± 0.1 mg/dl, and uric acid 4.7 ± 1.1 mg/dl. As for lipids, total cholesterol averaged 172.1 ± 41.8 mg/dl, and TG averaged 121.2 ± 63.5 mg/dl. HDL-c levels were 51.6 ± 9.7 mg/dL, while LDL-c reached 106.6 ± 40.8 mg/dL. Albumin was reported at 4.3 ± 0.3 g/dl. Liver function tests showed that ALT averaged 24.2 ± 17.5 U/L and AST was 24.3 ± 15.1 U/L. GGT values were 48.4 ± 86.3 U/L. As for CRP, an average of 9.9 ± 10.5 mg/L was found. Blood counts showed leukocytes at 6.2 ± 1.9 mil/mm^3^, erythrocytes at 4.6 ± 0.5 million/mm^3^, Hgb at 13.8 ± 2 g/dl, and hematocrit at 41.2 ± 5.1%. Finally, MCV was 88.9 ± 7.1 fL, HCM was 30 ± 3.1 pg, and platelets were recorded at 287 ± 98 mil/mm^3^. The average ESR was 17.2 ± 19.3 mm/h (Table 1).

### 3.3 Clinical and biochemical characteristics according to nutritional status

Table 2, In comparing clinical and biochemical characteristics between MN and well-nourished (WN) patients according to the CONUT and GLIM criteria, no statistically significant differences were identified in sex, age, disease duration, extent of disease according to the Montreal classification, gastrointestinal symptoms, complications, age at diagnosis, or disease duration between MN and WN patients using either assessment tool.

**TABLE 2:** Clinical and biochemical characteristics according to nutritional status assessed by CONUT and GLIM criteria.

	CONUT			GLIM		
Characteristic	MN *n* = 23	WN *n* = 28	*P*-value	MN *n* = 15	WN *n* = 36	*P*-value
Gender, n (%)						
Women	13 (57)	17 (61)	0.762	9 (60)	21 (58)	0.912
Men	10 (43)	11 (39)		6 (40)	15 (42)	
Age, years	41 ± 15	43 ± 14	0.655	39 ± 16	43 ± 14	0.299
Age of diagnosis, years	35 ± 17	36 ± 15	0.821	31 ± 18	37 ± 15	0.273
Disease evolution, years median (IQR)	3 (23)	4 (27)	0.682	5 (23)	3 (27)	0.736
Disease activity, *n* (%) Active	13 (57)	10 (35)	0.137	12 (80)	11 (30)	**0.001**
Remission	10 (43)	18 (65)		3 (20)	25 (70)	
Extension disease, n (%)						
Proctitis	4 (17)	4 (14)	0.389	2 (13)	6 (17)	0.958
Left colitis	3 (13)	9 (32)		3 (20)	9 (25)	
Pancolitis	15 (66)	13 (47)		9 (60)	19 (52)	
Uncertain	1 (4)	2 (7)		1 (7)	2 (6)	
Blood in stool, *n* (%)	4 (17)	6 (21)	0.371	3 (20)	7 (19)	0.549
Stool mucus, *n* (%)	8 (39)	7 (25)	0.287	6 (40)	10 (28)	0.248
Energy intake adequacy, % median (IQR)	101.4 (215.2)	86 (162)	0.655	92.1 (215.2)	94.7 (148)	0.991
ALMI, kg/m2 (DXA)						
Women	5.9 ± 1.2	6.4 ± 0.8	0.246	5.3 ± 0.9	6.6 ± 0.7	**0.001**
Men	7.6 ± 0.9	8.1 ± 0.9	0.458	7.1 ± 0.6	8.1 ± 0.9	**0.025**
Fat, % (BIA)	35 ± 10.5	33.7 ± 8.1	0.609	31.4 ± 10.3	35.6 ± 8.5	0.139
SMM, kg (BIA)	18.2 ± 4.7	20.2 ± 5.7	0.191	16.6 ± 4.8	20.4 ± 5.2	**0.019**
PhA, degrees	6 ± 0.7	6.5 ± 0.8	**0.026**	5.9 ± 0.9	6.4 ± 0.7	**0.035**
Albumin, g/dl	4.2 ± 0.3	4.3 ± 0.2	0.156	4.2 ± 0.3	4.3 ± 0.3	0.182
Total cholesterol, mg/dl	140.6 ± 24	198.1 ± 35.3	**<0.001**	163.4 ± 39.1	175.8 ± 42.9	0.341
Lymphocytes cel/μl	1.6 ± 0.7	1.9 ± 0.4	0.061	1.5 ± 0.5	1.9 ± 0.6	**0.050**
Hemoglobin, g/dl	12.9 ± 2.4	14.6 ± 1.1	**0.004**	13.2 ± 2.3	14.1 ± 1.8	0.168
Triglycerides, mg/dl	98.3 ± 48.9	140.7 ± 68.7	**0.017**	115.7 ± 62.9	123.5 ± 64.5	0.694

MN, malnutrition; WN, well-nourished; CONUT, Controlling Nutritional Status; GLIM, Global Leadership Initiative on Malnutrition; DXA, dual-energy X-ray absorptiometry; BIA, bioelectrical impedance analysis; SMM, skeletal muscle mass; PhA, phase angle; ALMI, appendicular lean mass index. Continuous variables as median ± standard deviation (SD). Energy intake adequacy and disease evolution is presented as median (interquartile range, IQR). The *t*-test was used for continuous variables, and the chi-square (χ^2^) test for categorical variables, *p* < 0.05 was considered statistically significant. Bold values indicate statistically significant differences (*p* < 0.05).

When comparing patients classified as MN by CONUT and GLIM, total cholesterol levels were significantly lower in patients with CONUT MN (140.6 ± 24 vs. 163.4 ± 39.1 mg/dL, *p* = 0.031). No other significant differences were observed between MN groups.

Differences between patients with MN and WN varied across assessment methods. When evaluated with CONUT, patients with MN showed significantly lower PhA (6° ± 0.7 vs. 6.5° ± 0.8, *p* = 0.026), total cholesterol levels (140.6 ± 24 vs. 198.1 ± 35.3 mg/dl, *p* < 0.001), Hgb (12.9 ± 2.4 vs. 14.6 ± 1.1 g/dl, *p* = 0.004) and TG (98.3 ± 48.9 vs. 140.7 ± 68.7 mg/dl, *p* = 0.017) compared with WN patients. And the evaluation with GLIM criteria, we found in higher proportion of active disease (*p* = 0.001), and lower lymphocyte count (1.5 ± 0.5 vs. 1.9 ± 0.6 cells/μL, *p* = 0.050), ALMI in women (5.3 ± 0.9 vs. 6.6 ± 0.7 kg/m^2^, *p* = 0.001), ALMI in men (7.1 ± 0.6 vs. 8.1 ± 0.9 kg/m^2^, *p* = 0.025), SMM by BIA (16.6 ± 4.8 vs. 20.4 ± 5.2 kg, *p* = 0.019), and PhA (5.9 ± 0.9 vs. 6.4 ± 0.7°, *p* = 0.035) compared with WN patients.

Energy intake and energy adequacy were comparable between MN and WN patients, regardless of the nutritional assessment method used. The median energy adequacy ranged from approximately 86%–101% of estimated needs across all groups. Few patients had an energy intake below 50% of estimated needs.

### 3.4 Nutritional status and diagnostic performance according to GLIM and CONUT

According to the GLIM criteria, 15 patients (29%) were classified as MN, whereas 23 patients (45%) were classified as MN by the CONUT score. Both methods concorded MN in 11 patients, while 24 patients were consistently classified as WN by GLIM and CONUT, with an area under the curve (AUC) of 0.705 (95% CI: 0.537–0.872).

Phenotypic GLIM criteria included weight loss in 12 patients (23.5%), low BMI in 7 (13.7%), and reduced muscle mass in 12 (23.5%). Among the etiologic criteria, reduced energy intake was identified in 3 of 48 patients (6.3%), while active disease was present in 23 (45.1%); therefore, GLIM classification was predominantly driven by disease activity and/or inflammation.

The sensitivity and specificity of the diagnostic test were calculated as follows: sensitivity, 73%; specificity, 67%. The positive and negative predictive values were 48% and 86%, respectively (Table 3).

**TABLE 3:** Diagnostic accuracy of CONUT and CONUT-derived scores for identifying GLIM-defined MN.

Score	Sensitivity (%)	Specificity (%)	PPV (%)	NPV (%)	Youden index	*P*-value	Kappa	AUC	95% CI
CONUT	73	67	48	86	0.40	0.010	0.346	0.705	0.537–0.872
eCONUT-UC-PhA	80	57	44	87	0.37	0.016	0.302	0.741	0.587–0.895
eCONUT-UC-Hgb	80	64	48	88	0.44	0.005	0.367	0.738	0.586–0.890
eCONUT-UC-TG	73	51	39	82	0.25	0.095	0.198	0.642	0.472–0.812

CONUT, Controlling Nutritional Status; eCONUT, modified CONUT; MN, malnutrition; PhA, phase angle; Hgb, hemoglobin; TG, triglycerides; AUC, area under the ROC curve. Sensitivity, specificity, positive predictive value (PPV), and negative predictive value (NPV), confidence interval (CI) were calculated using GLIM-defined malnutrition as the reference standard. Analyses included *n* = 51 for CONUT and eCONUT-UC-Hgb and *n* = 50 for eCONUT-UC-PhA and eCONUT-UC-TG because of one missing PhA value and one missing TG value, respectively.

### 3.5 Diagnostic accuracy of the eCONUT-UC score

The modified versions, eCONUT-UC-PhA and eCONUT-UC-Hgb, showed higher sensitivity (80%) than the original CONUT, with specificities of 57% and 64%, respectively, and AUCs of 0.741 (95% CI: 0.587–0.895) and 0.738 (95% CI: 0.586–0.890). In contrast, eCONUT-UC-TG showed lower diagnostic performance, with a sensitivity of 73%, a specificity of 51%, and an AUC of 0.642 (95% CI: 0.472–0.812), compared with its other modifications (Figure 2).

**FIGURE 2:**
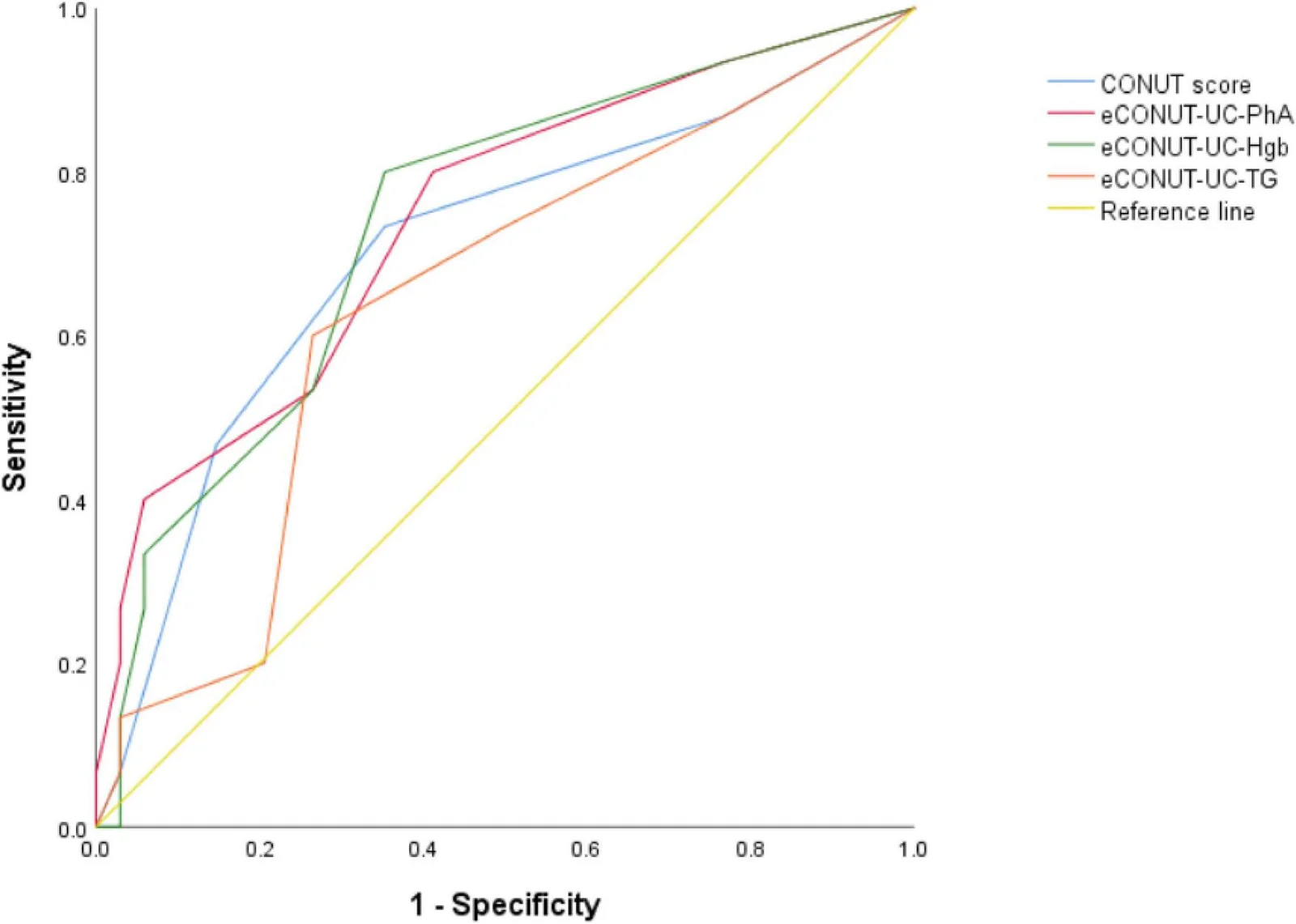
ROC curves: The diagnostic performance of CONUT and its enhanced versions (eCONUT-UC-PhA, eCONUT-UC-Hgb, and eCONUT-UC-TG) is shown for identifying MN defined according to GLIM criteria. CONUT, Controlling Nutritional Status; MN, malnutrition; eCONUT, enhanced CONUT; PhA, phase angle; Hgb, hemoglobin; TG, triglycerides; GLIM, Global Leadership Initiative on Malnutrition; ROC, receiver operating characteristic.

Pairwise comparison of the ROC curves using DeLong’s test showed no statistically significant differences in AUC between the original CONUT and eCONUT-UC-PhA (*p* = 0.922) or eCONUT-UC-Hgb (*p* = 0.799), indicating that the numerical increases in AUC did not represent a statistically significant improvement in discrimination.

The Youden index was higher for eCONUT-UC-Hgb (0.44) than for CONUT (0.40), eCONUT-UC-PhA (0.37), and eCONUT-UC-TG (0.25), indicating better overall diagnostic performance of the hemoglobin-based model.

Statistically significant associations with MN as defined by GLIM criteria were found for CONUT (*p* = 0.010), eCONUT-UC-PhA (*p* = 0.016), and eCONUT-UC-Hgb (*p* = 0.005). However, the association for eCONUT-UC-TG was not significant (*p* = 0.095), and as for agreement with the GLIM classification, the kappa coefficient was *k* = 0.302 for eCONUT-UC-PhA, and *k* = 0.367 for eCONUT-UC-Hgb (Table 3).

## 4 Discussion

The eCONUT-UC was developed as an improved score for detecting MN in patients with UC. This version includes variables that reflect different aspects of nutritional status. This is of paramount importance in clinical practice, as timely nutritional intervention in UC patients can improve disease prognosis.

Assessing nutritional status in patients with IBD remains challenging; in recent years, the GLIM criteria have been proposed and consolidated as an international consensus framework for diagnosing MN (16). Several studies have evaluated the application of GLIM criteria in patients with IBD, reported a considerable prevalence of MN, and highlighted the limitations of traditional screening tools relative to GLIM-defined MN. Therefore, our study used the GLIM criteria to define MN as the reference standard for evaluating the diagnostic performance of CONUT and the eCONUT-UC variants in patients with UC.

Our findings showed that the rate of MN in patients with UC was 29%. Previous studies have reported a higher prevalence of MN in combined IBD. For example, Zhang et al., included 238 patients with IBD (177 with Crohn’s disease and 61 with UC) and found a high prevalence of 60.1% according to the GLIM criteria (2). Similar results were reported by Fiorindi et al. (17), and 40% of patients had MN according to the GLIM criteria. Other studies, such as Liu’s, found that patients with Crohn’s disease (CD) have higher MN levels, up to 57%, compared with 38.8% in patients with UC. Disease activity, quality of life, and increased perceived stress are associated with MN (18). More recently, Korwel et al. evaluated 306 outpatients with IBD, reporting a prevalence of 11.8% according to GLIM, identifying that women and disease activity were independently associated with the risk of MN (19).

Much of the evidence includes mixed populations of IBD, with a higher proportion of patients with CD, which is a presentation of IBD that is associated with a higher risk of MN, due to its pathophysiology, which is characterized by greater inflammatory load and malabsorption. This explains the differences in MN rates compared with ours, which focuses exclusively on patients with UC.

Although GLIM is a comprehensive tool for detecting MN, its applicability may be limited by its inability to meet all its criteria. Ideally, its application requires specialized nutritional equipment to obtain 24R and body composition equipment such as BIA or DXA, which could be difficult to access in some health centers (12). We present reliable alternatives that can be easily applied across different health centers, depending on resource availability.

Our research team considered the CONUT a possible tool because of its simplicity and quantitative format, which facilitates interpretation and does not require validation across languages, making it a reliable tool for clinical application.

In a study by de León-Rendón et al., higher CONUT scores were associated with increased disease activity and inflammatory markers, such as CRP and ESR, highlighting its potential role not only in nutritional screening but also in disease severity. These findings support the potential utility of CONUT as a simple, accessible screening instrument in clinical practice, particularly in settings where rapid laboratory tools are needed to identify patients at nutritional risk (20).

However, the CONUT score is based only on biochemical parameters, which may not fully capture the multidimensional nature of MN; therefore, it was decided to explore another quantitative variable that can be obtained in the assessment of nutritional status, whether anthropometric, biochemical, clinical, or dietetic, and to strengthen its diagnostic performance.

Among the variables we identified for this objective, we also identified other biochemical variables, such as Hgb and TG, which is logical, given that both have been associated with nutritional status and disease activity in patients with IBD.

Anemia is one of the most prevalent complications in this population; it is usually related to nutritional deficiencies, chronic inflammation, and intestinal malabsorption (21). As shown by Koutroubakis et al., who included 410 patients with IBD, persistent or recurrent anemia was associated with more aggressive and disabling disease, higher frequency of hospitalizations, and lower quality of life compared to those without anemia (22). Similar results were reported by Dutta et al., they analyzed a cohort of 1763 patients with IBD and observed that 54% had anemia, which was associated with more hospitalizations, longer hospital stays, and a greater number of medical consultations (23). On the other hand, Bergamaschi et al., found a prevalence of anemia of 13.6%, being more frequent in women and associated with greater fatigue and poorer quality of life. Iron deficiency was the most frequent cause of anemia, representing 62.5% of cases, highlighting the close relationship among anemia, inflammation, and nutritional status in this population (24). On the other hand, low triglyceride levels have been associated with a higher risk of rehospitalization or death, suggesting that this finding could reflect underlying frailty, MN, and inflammatory burden. Alterations in lipid metabolism could also reflect disease severity and underlying metabolic disorders, as demonstrated by Soh et al., who observed that low TG levels were associated with a higher incidence of UC (25), suggesting that lipid metabolism may be linked to the disease’s pathophysiology. Other studies have shown that low cholesterol, HDL-c, and LDL-c levels correlate with disease severity (26, 27). The authors infer that this association could reflect MN or an underlying disease.

On the other hand, the PhA has been proposed as a marker of cellular integrity and nutritional status, based on the BIA analysis. Lower PhA values have been associated with inflammation, poorer cellular health, and poorer nutritional status in patients with IBD (28). Previous research has suggested that PhA can serve as a complementary tool for nutritional assessment in patients with UC, supporting its potential integration into screening approaches that seek to improve the early identification of MN, as shown Bongiolo et al., observed that there is a significant correlation with SMM (rs = 0.35, *p* < 0.001), identified PhA cut-off points ≤5.04° in women and ≤6.08° in men to predict muscle mass reduction (29). Similarly, Mendoza-Martínez et al. (28) evaluated the PhA in patients with UC and found it was significantly lower than in healthy subjects (5.8 ± 0.8° vs. 6.6 ± 0.7°, *p* < 0.001). In addition, it was associated with a lower SMM, lower ICW, and higher ECW. Likewise, PhA cut-off points <6.1° in men and <5.6° in women were proposed to identify nutritional compromise in patients with UC. These findings support the use of PhA as a useful, non-invasive tool for early detection of MN and alterations in body composition in patients with IBD. This is consistent with our findings, which show that PhA was significantly lower in patients classified as MN compared to WN individuals, regardless of the assessment tool used. The usefulness of PhA as a non-invasive indicator of early MN detection and related changes in body composition, particularly the loss of SMM in patients with UC, is evident.

The proposed cut-off points were derived not only from statistical optimization but also selected for their clinical relevance. From a clinical perspective, these thresholds aim to identify early alterations in nutritional status, which may precede over MN.

Studies still indicate that MN remains underdiagnosed in clinical practice, despite its high prevalence and clinical impact (30, 31). A qualitative study with physicians’ staff highlights that MN is often assessed not with standardized tools but through clinical judgment, and its identification and management are often delegated to dietitians. Identified barriers include lack of time, staff shortages, limited access to dietitians, and a lack of resources for comprehensive nutritional assessment (31).

In this context, eCONUT-UC addresses these limitations by providing a fast and accessible tool that improves early detection of MN in patients with UC and can be used not only by dietitians but also by physicians.

In the study by Schuetz et al., early, individualized nutritional support was shown to reduce adverse clinical outcomes (23% vs. 27%; adjusted OR 0.79, 95% CI 0.64–0.97; *p* = 0.023) and mortality (7% vs. 10%; adjusted OR 0.65, 95% CI 0.47–0.91; *p* = 0.011) in patients at nutritional risk. These findings confirm the need for a systematic assessment of nutritional risk in hospitalized patients upon admission, regardless of their medical condition, followed by a nutritional evaluation and the implementation of personalized nutritional support (32).

Our findings showed low agreement between classifications derived from eCONUT-UC and GLIM criteria (*k* < 0.40). However, similar findings have been reported, where diagnostic criteria such as GLIM and ESPEN show little agreement. This may reflect differences in the conceptualization of MN and in the tools’ ability to detect early or subclinical stages that do not yet meet diagnostic criteria. A recent prospective study that assessed both the GLIM and the SGA found low agreement with the ESPEN definition of MN (*k* < 0.40), highlighting variability across diagnostic criteria and the lack of a universally accepted standard for diagnosing MN (33).

The eCONUT-UC could be easily implemented during routine laboratory evaluations, allowing physicians to quickly identify patients with MN without specialized equipment. Further studies focusing on external validation are needed.

## 5 Limitations

We acknowledge several limitations of our study. First, we assessed dietary intake using a single 24-h recall, which may not accurately reflect usual intake and is subject to recall bias. The cross-sectional design limits our ability to establish causal relationships.

The eCONUT-UC versions and their corresponding cut-off points were derived from a relatively small Mexican population specific to UC, which may limit the generalizability of the findings. Furthermore, only outpatients were included, so the findings cannot be generalized to hospitalized patients or other clinical settings.

In addition, given the exploratory nature of the study, the proposed cut-off points require external validation in independent cohorts. These variants were not compared with other tools such as the MUST. Moreover, the study did not assess the association between the CONUT variants and clinical outcomes.

Because the eCONUT-UC variants were developed as *post hoc* exploratory analyses, the original sample size calculation was not specifically powered for their development or comparison. In addition, only 15 participants met the GLIM criteria for MN, resulting in limited precision of the diagnostic performance estimates.

Additionally, selection bias may be present because the study was conducted at a single tertiary-care center, which may limit the generalizability of the findings. Information bias may also be present due to the use of clinical records and 24R data.

Furthermore, because the cut-off points and diagnostic performance of the eCONUT-UC variants were derived and evaluated in the same cohort, the reported sensitivity, specificity, and AUC values represent apparent performance and may be optimistically biased. No internal optimism correction was performed in the present exploratory analysis. Therefore, these results should be interpreted as hypothesis-generating estimates, and internal and external validation are required before the proposed scores can be recommended for clinical use.

## 6 Conclusion

Phase angle levels were significantly lower in patients with MN, regardless of the assessment tool used, supporting its potential role as a marker of nutritional status. Furthermore, alterations in the lipid profile were observed in patients classified as MN by CONUT; however, these differences were not significant when MN was defined using the GLIM criteria.

Our findings support eCONUT-UC-Hgb and eCONUT-UC-PhA as promising candidate modifications of the CONUT score for further validation in UC. However, because the cut-offs and diagnostic performance were derived within the same cohort, these findings should be considered exploratory and require validation before clinical implementation.

Among the variants evaluated, eCONUT-UC-Hgb could represent the most practical candidate for future validation, as it relies exclusively on routinely available laboratory parameters and showed the highest Youden index and the highest concordance with GLIM criteria among the improved variants.

The rate of MN varied by assessment tool, with higher rates observed with CONUT than with GLIM (45% vs. 29%). This discrepancy suggests that the tools may reflect different dimensions or stages of nutritional impairment.

A higher number of patients with active disease were found among those classified as having MN using GLIM, supporting the association between inflammation and nutritional impairment in UC.

Malnutrition in UC is a multifactorial condition, characterized not only by changes in dietary intake but also by alterations in body composition and biochemical parameters.

## Data Availability

The raw data supporting the conclusions of this article will be made available by the authors, without undue reservation.
