# Supplementary material for: eCONUT-UC for nutritional screening and malnutrition identification in ulcerative colitis
Source: Front Nutr. 2026 Sep 24;13:1907895. doi: 10.3389/fnut.2026.1907895 (PMC13645595; doi:10.3389/fnut.2026.1907895)
Supplement: Supplementary file 1 [file Table_1.docx]

Supplementary Material

| Table 1. Application of GLIM criteria | | | | |
| --- | --- | --- | --- | --- |
| **Phenotypic Criteria** | | | **Etiological criteria** | |
| **Weight loss** | **BMI** | **Muscle Mass Reduction** | **Decreased dietary intake or assimilation** | **Inflammation** |
| >5% in 6 months | <20 in 70 years | <7kg/m^2^ is considered low muscle mass for men and <5.5kg/m^2^ was considered low muscle mass for women | <50% of the ER in >1 week, according to 24R or patients with TLW criteria positive | Chronic inflammatory pathology with a CRP >5mg/L |
| GLIM: Global Leadership Initiative on Malnutrition, BMI: Body Mass Index, ER: Energy Requirement, TLW: Truelove and Witts, CRP: C-reactive Protein, kg/m2:kilograms per square meter, 24-hour dietary recalls: 24R. Reference: Cederholm T. *et al.*, 2019 (3). | | | | |

| **Table 2. CONUT Score** | | | | |
| --- | --- | --- | --- | --- |
|  | **Normal** | **Mild** | **Moderate** | **Severe** |
| Albumin g/dl | >3.5  (0) | 3-3.49  (2) | 2.5-2.99  (4) | <2.5  (6) |
| Total Cholesterol mg/dl | >180  (0) | 140-179  (1) | 100-139  (2) | <100  (3) |
| Lymphocytes cel/µl | >1600  (0) | 1200-1599  (1) | 800-1199  (2) | <799  (3) |
| CONUT (Controlling Nutritional Status), mg/dl (milligram per deciliter), g/dl (gram per deciliter), cel/µl (cells per microliter), Reference: García-Vázquez N. *et al.,*  2020 (6) | | | | |

| **Table 3. Scoring of enhanced Controlling Nutritional Status score for Ulcerative Colitis** | | | | |
| --- | --- | --- | --- | --- |
|  | **Normal** | **Mild** | **Moderate** | **Severe** |
| **Option 1: eCONUT-UC-PhA** | | | | |
| PhA, degrees | ≥6  (0) | ≥ 5.6 - <6  (1) | ≥ 5.0 - <5.6  (2) | ≤5  (3) |
| **Option 2: eCONUT-UC-Hgb** | | | | |
| Hemoglobin, g/dl | >13  (0) | 12.0-12.9  (1) | 10.5-11.9  (2) | <10.4  (3) |
| **Option 3: eCONUT-UC-TG** | | | | |
| TG, mg/dl | >80  (0) | 65-79  (1) | 50-64  (2) | <49  (3) |
| eCONUT-UC (enhanced Controlling Nutritional Status score for Ulcerative Colitis). Each option represents an alternative CONUT-derived score, calculated by adding only one parameter (PhA: phase angle, Hgb: hemoglobin, or TG: triglycerides) to the original CONUT score. The three options must be evaluated independently and not combined into a single composite score. | | | | |

| **Table 4. Contingency tables for CONUT and eCONUT-UC variants against GLIM-defined malnutrition** | | | | | |
| --- | --- | --- | --- | --- | --- |
| **Test** | **TP** | **FP** | **FN** | **TN** | **Total** |
| CONUT | 11 | 12 | 4 | 24 | 51 |
| eCONUT-UC-PhA | 12 | 15 | 3 | 20 | 50 |
| eCONUT-UC-Hgb | 12 | 13 | 3 | 23 | 51 |
| eCONUT-UC-TG | 11 | 17 | 4 | 18 | 50 |
| TP, true positive; FP, false positive; FN, false negative; TN, true negative; GLIM, Global Leadership Initiative on Malnutrition. | | | | | |

**Figure 1.** STARD Statement—Checklist of items that should be included in reports of **diagnostic accuracy study**

|  | **Section & Topic** | **No** | **Item** | **Reported on page #** |
| --- | --- | --- | --- | --- |
|  |  |  |  |  |
|  | **TITLE OR ABSTRACT** |  |  |  |
|  |  | **1** | Identification as a study of diagnostic accuracy using at least one measure of accuracy  (such as sensitivity, specificity, predictive values, or AUC) | 1 |
|  | **ABSTRACT** |  |  |  |
|  |  | **2** | Structured summary of study design, methods, results, and conclusions  (for specific guidance, see STARD for Abstracts) | 2 |
|  | **INTRODUCTION** |  |  |  |
|  |  | **3** | Scientific and clinical background, including the intended use and clinical role of the index test | 3-4 |
|  |  | **4** | Study objectives and hypotheses | 4 |
|  | **METHODS** |  |  |  |
|  | *Study design* | **5** | Whether data collection was planned before the index test and reference standard  were performed (prospective study) or after (retrospective study) | 4 |
|  | *Participants* | **6** | Eligibility criteria | 4 |
|  |  | **7** | On what basis potentially eligible participants were identified  (such as symptoms, results from previous tests, inclusion in registry) | 4 |
|  |  | **8** | Where and when potentially eligible participants were identified (setting, location and dates) | 4 |
|  |  | **9** | Whether participants formed a consecutive, random or convenience series | 4 |
|  | *Test methods* | **10a** | Index test, in sufficient detail to allow replication | 7 |
|  |  | **10b** | Reference standard, in sufficient detail to allow replication | 6 |
|  |  | **11** | Rationale for choosing the reference standard (if alternatives exist) | 3-4 |
|  |  | **12a** | Definition of and rationale for test positivity cut-offs or result categories  of the index test, distinguishing pre-specified from exploratory | 7 |
|  |  | **12b** | Definition of and rationale for test positivity cut-offs or result categories  of the reference standard, distinguishing pre-specified from exploratory | 7 |
|  |  | **13a** | Whether clinical information and reference standard results were available  to the performers/readers of the index test | 6 |
|  |  | **13b** | Whether clinical information and index test results were available  to the assessors of the reference standard | 6 |
|  | *Analysis* | **14** | Methods for estimating or comparing measures of diagnostic accuracy | 7-8 |
|  |  | **15** | How indeterminate index test or reference standard results were handled | 7-8 |
|  |  | **16** | How missing data on the index test and reference standard were handled | 8 |
|  |  | **17** | Any analyses of variability in diagnostic accuracy, distinguishing pre-specified from exploratory | 8 |
|  |  | **18** | Intended sample size and how it was determined | 7-8 |
|  | **RESULTS** |  |  |  |
|  | *Participants* | **19** | Flow of participants, using a diagram | 8 |
|  |  | **20** | Baseline demographic and clinical characteristics of participants | 8 |
|  |  | **21a** | Distribution of severity of disease in those with the target condition | 9 |
|  |  | **21b** | Distribution of alternative diagnoses in those without the target condition | 9 |
|  |  | **22** | Time interval and any clinical interventions between index test and reference standard | 9-10 |
|  | *Test results* | **23** | Cross tabulation of the index test results (or their distribution)  by the results of the reference standard | 10 |
|  |  | **24** | Estimates of diagnostic accuracy and their precision (such as 95% confidence intervals) | 10 |
|  |  | **25** | Any adverse events from performing the index test or the reference standard | NA |
|  | **DISCUSSION** |  |  |  |
|  |  | **26** | Study limitations, including sources of potential bias, statistical uncertainty, and generalisability | 11-15 |
|  |  | **27** | Implications for practice, including the intended use and clinical role of the index test | 11-15 |
|  | **OTHER INFORMATION** |  |  |  |
|  |  | **28** | Registration number and name of registry | 4 |
|  |  | **29** | Where the full study protocol can be accessed | 16 |
|  |  | **30** | Sources of funding and other support; role of funders | 16 |
|  |  |  |  |  |
